# Lung Ultrasound: Its Findings and New Applications in Neonatology and Pediatric Diseases

**DOI:** 10.3390/diagnostics11040652

**Published:** 2021-04-03

**Authors:** Elio Iovine, Raffaella Nenna, Silvia Bloise, Domenico Paolo La Regina, Daniela Pepino, Laura Petrarca, Antonella Frassanito, Riccardo Lubrano, Fabio Midulla

**Affiliations:** 1 Department of Maternal Infantile and Urological Sciences, Sapienza University of Rome, 00161 Rome, Italy; elio.iovine@gmail.com (E.I.); laregina.domenico@gmail.com (D.P.L.R.); danpepino@hotmail.com (D.P.); laurapetrarca85@gmail.com (L.P.); antonellafras@libero.it (A.F.); midulla@uniroma1.it (F.M.); 2 Pediatric and Neonatology Unit, Maternal and Child Department, Sapienza University of Rome, Polo Pontino, 4100 Latina, Italy; silvia.bloise1989@gmail.com (S.B.); riccardo.lubrano@uniroma1.it (R.L.)

**Keywords:** lung ultrasound, infants, children, pneumonia, bronchiolitis, COVID-19

## Abstract

Lung ultrasound has become increasingly used in both adult and pediatric populations, allowing the rapid evaluation of many lung and pleura diseases. This popularity is due to several advantages of the method such as the low cost, rapidity, lack of ionizing radiation, availability of bedside and repeatability of the method. These features are even more important after the outbreak of the SARS-CoV-2 pandemic, given the possibility of recognizing through ultrasound the signs of interstitial lung syndrome typical of pneumonia caused by the virus. The purpose of this paper is to review the available evidence of lung ultrasound (LUS) in children and its main applications in pediatric diseases.

## 1. Introduction

The management of pediatric lung diseases has always been challenging for clinicians because of the variable clinical manifestations and often overlapping symptoms and signs. Traditionally, chest X-ray (CXR) has played a crucial role in the diagnosis of respiratory diseases. However, the potentially harmful effects of radiation exposure due to CXR reduce its applications, especially in children [1].

In recent years, a new imaging application of sonography has spread in emergency departments and clinical practice: lung ultrasound (LUS) [2]. It is quick, portable, repeatable, lacks ionizing radiation and, therefore, finds application in many different settings, both inpatient and outpatient, in both acute and chronic conditions [3]. In particular, growing evidence highlights the effectiveness of this method as a diagnostic tool in different pediatric diseases [4,5]. Infants and children are considered ideal candidates for this type of exam due to their thinner chest and smaller lung volumes. These features help to make any lesions better visible because, in smaller lungs, it is more likely that such lesions reach the pleura and make the linear probe able to complete the exam [6].

Moreover, in the era of the coronavirus disease 2019 (COVID-19) pandemic, lung ultrasound has further proven its potential, finding application in early diagnosis, follow-up and management of patients affected by SARS-CoV-2 infection [7,8].

However, this method has some limitations. In fact, LUS is an operator-dependent examination, and the best performance is mainly obtained with experienced operators trained in pulmonary ultrasound. In addition, ultrasound is only able to detect lesions if directly reaching the pleura, with no normally aerated lung between the pleura and the lesion [9].

The purpose of this paper is to review the available evidence of LUS in children and its main applications in pediatric diseases.

## 2. Technique and Equipment

Children can be scanned in the upright, supine or lateral decubitus position; furthermore, to obtain major compliance in noncollaborating children, the exam can be performed with a warmed-up gel and the child seated in the caregiver’s lap during breastfeeding or by using age-appropriate distraction techniques in order to minimize anxiety.

A linear probe of 10 Mhz is the most commonly used transducer for LUS examination [5]. This type of transducer, given the use of high frequencies, is optimal for investigating up to about 4 cm in depth of the chest and then accurately visualizing especially the pleural line and its possible alterations. Instead, lower-frequency probes, such as the curvilinear transducer (1–5 MHz) or the phased-array transducer (1–3 MHz), ensure higher and wider penetration, useful for a general evaluation of the lung or in the suspect of diffuse disease with a lower resolution than that of high-frequency linear probes.

The compound should also be turned off because the B-lines disappear when the pleura is insonified from different angles, as done in compound imaging. This will cancel the reverberation artifact, reducing the brightness of B-lines.

Each hemithorax is divided into three areas: (1) anterior area, delimited by parasternal and anterior axillary lines; (2) lateral area, within the anterior and posterior axillary lines; and (3) posterior area beyond the posterior axillary line. Every area is subdivided into upper and lower halves.

The probe is placed vertically, obliquely and horizontally to the ribs in the anterior, lateral and posterior thorax and moved from one intercostal space to the next, in a caudal direction, from the apices to the costophrenic angles in order to cover the entire lung surface [6]. In all cases, this systematic approach to lung exploration should be used, especially at the patient’s first imaging examination. A focused approach can then be applied if signs of disease have been identified by the first scans or by another imaging method.

To date, several scoring systems have been proposed to assess the degree of lung involvement in different lung diseases to evaluate the distribution of B-lines, one of the most common ultrasound artifacts. These systems divide the thorax into a different number of zones and assign a score based on the distribution of B-lines in the different explored areas during the scan. Among these, the most frequently used in critical care settings are the 4-zone, 6-zone and 8-zone systems introduced by Platz et al. [10], the 12-zone system by Volpicelli et al. [3] and the 14-zone system by Soldati et al. [11].

As recently observed [12], the timing of performance of lung ultrasound is a pivotal element to be evaluated in order to use the full potential of the method. At the moment, there are no studies that directly analyze the impact of the timing of execution in some clinical conditions on its accuracy, but it seems reasonable that ultrasound should be performed as soon as possible or at least within 24 h of admission to the hospital in the case of infectious diseases [13].

## 3. Lung Ultrasound Findings in Healthy Subjects

The first layers of the chest consist of subcutaneous tissue and muscles. In longitudinal sections, the ribs appear as curved structures with a posterior acoustic shadow. In axial sections, the intercostal acoustic window is used to study tissues located deep into the skin and muscle [14]. In a normally aerated lung, the only detectable structure is the pleura. It appears as a continuous hyperechogenic line that moves back and forth with the breaths. This movement is also known as “lung sliding” [15].

Below the pleural line, the lung is filled with air. This does not allow the direct visualization of the normal pulmonary parenchyma, but some artifacts and echographic findings remain describable.

**Figure 1:**
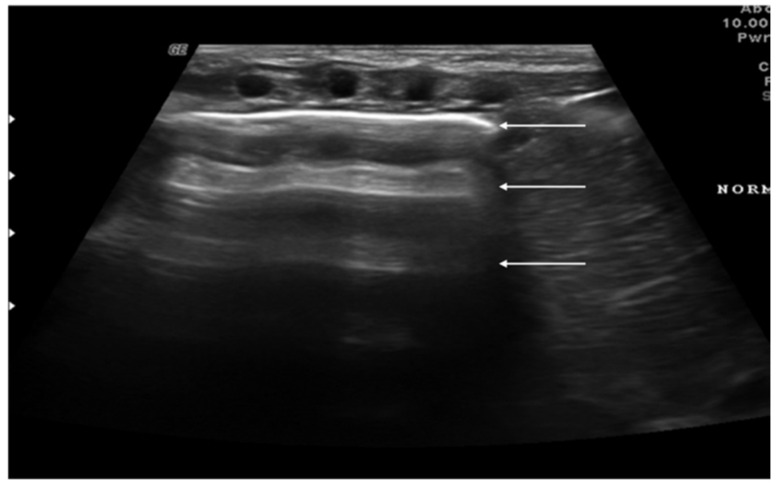
Appearance of a healthy lung. White arrows show pleural and A-lines.

“A-lines” represent some of these artifacts that can be found in a completely healthy lung (Figure 1) [14]. They are horizontal echogenic lines equidistant and parallel to each other and the pleura, representing reverberations of the pleura itself that arise when the ultrasound beam reflects off of the pleura and partially reflects off of the probe face back to the pleura again before getting back to the machine instead of entering the probe. They are caused by the large difference in acoustic impedance between the pleura and the air contained in the lungs. The distance from each other is related to the distance between the pleural line and the skin surface, and their position does not change with respiratory acts.

**Figure 2:**
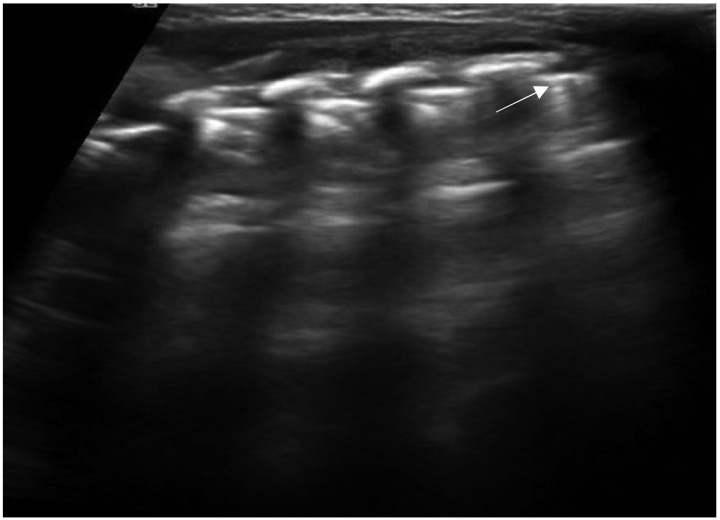
Isolated Z-lines (white arrow).

**Figure 3:**
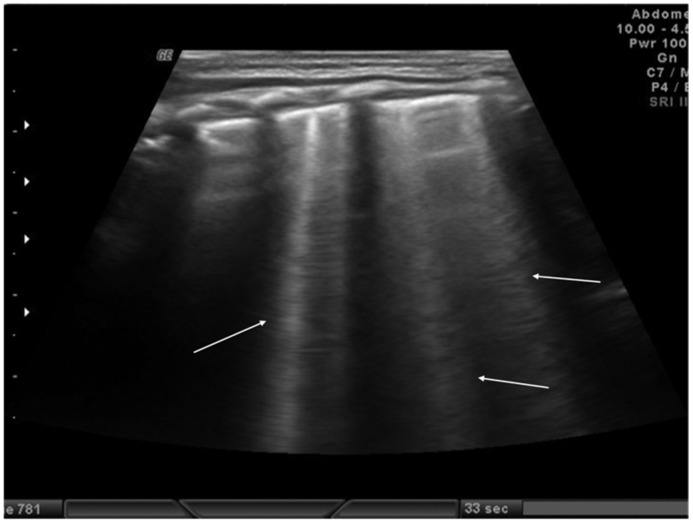
Isolated B-lines (white arrows).

Another sonographic artifact is represented by the “Z-lines”. These artifacts are formed by reverberation echoes from irregularities on the lung surface and appear as vertical echogenic lines (but less echogenic than a pleural line) originating from the pleura that does not erase the A-lines and do not move with lung sliding (Figure 2) [16]. These should be distinguished from B-lines that are similar to Z-lines but usually indicate the presence of fluid in the interstitial compartment or, in general, an abnormality in the alveolar or interstitial compartment. B-lines appear as a vertical line arising from the pleura, often erasing the A-lines and moving with the lung sliding (Figure 3) [16].

Although these artifacts are not present in a normal lung, they may be observed in the infant’s lung in the first 48 h of life because of the possible delay in lung fluid resorption [17].

Until 2020, pediatric studies of lung ultrasound were based on the assumption that the ultrasound appearance of a healthy adult lung was the same as that of a child, and no studies had ever been conducted to describe the appearance of the healthy lung in children, particularly infants. Recently, Buonsenso et al. described the normal ultrasound appearance of the lung in infants during the first 6 months of life by performing an ultrasound at 10 days and 1, 3 and 6 months. The study showed that in the first months of life the healthy infant’s lung is characterized by a B-pattern with multiple vertical artifacts that tend to normalize toward the normal A-pattern typical of the adults at six months of age. No consolidations, pleural line abnormalities or pleural effusion were observed in healthy infants [18].

## 4. Lung and Chest Wall Ultrasound Applications in Children

The main indications for lung and chest wall ultrasound in the pediatric population include:

- Diagnosis and follow-up of neonatal lung diseases;
- Confirm antenatal diagnosis of lung malformations;
- Diagnosis and follow-up of pediatric lung infectious diseases (namely bronchiolitis and pneumonia) and lung complications (such as pneumothorax, pleural effusion and lung abscess);
- Diagnosis and follow-up of pulmonary edema;
- Diagnosis of thoracic trauma and early detection of signs of child abuse;
- Follow-up in children undergoing cardiac surgery;
- Diaphragm ultrasound.

## 5. Neonatal Lung Diseases

Many lung diseases, such as respiratory distress syndrome, transient tachypnea, pneumonia, atelectasis and pneumothorax, can cause respiratory distress in newborns, especially among premature infants. The differential diagnosis of these diseases is often difficult, but LUS can help to discriminate these diseases [19,20].

**Figure 4:**
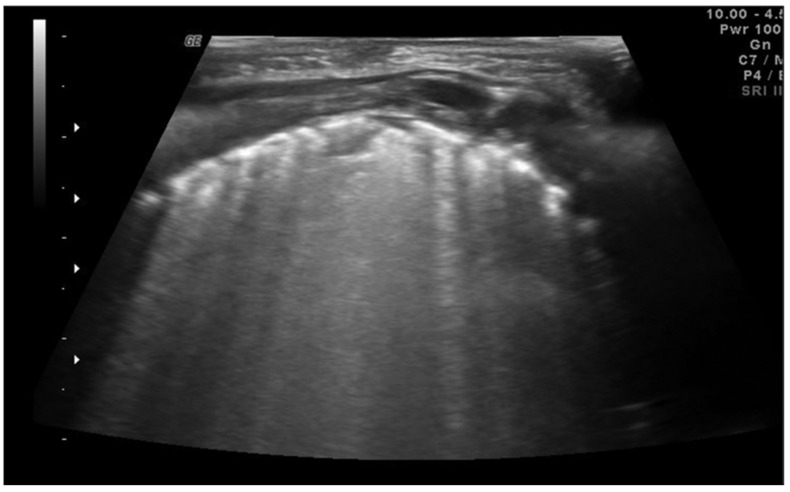
Neonatal respiratory distress syndrome (NRDS). Coalescent B-lines, irregularity of the pleural line and subpleural consolidation are visible.

Neonatal respiratory distress syndrome (NRDS) is characterized by a functional and structural immaturity of the lung resulting in respiratory distress that appears at birth. The ultrasound findings of NRDS are represented by coalescent B-lines, diffuse and symmetrically distributed in both lungs. These lines are due to the presence of fluid in the interstitial or alveolar compartment, and generally, their number correlates with the severity of the disease, up to “white lung” in the most severe cases. The pleural line appears irregular, poorly defined and thickened. In addition, small areas of hypoechogenic subpleural consolidation can often be observed, especially in the posterior lung fields (Figure 4) [21].

The 2019 European consensus guidelines on the management of respiratory distress syndrome assert that lung ultrasound could be a useful tool for clinical decision making, and it seems able to differentiate NRDS from other common respiratory disorders of the newborn, reducing exposure to ionizing radiation [22]. In addition, some recent evidence has underlined how ultrasound performed in the first 12 h of life can be useful in identifying patients most likely to require surfactant therapy or mechanical ventilation, even before the oxygenation criteria [23].

**Figure 5:**
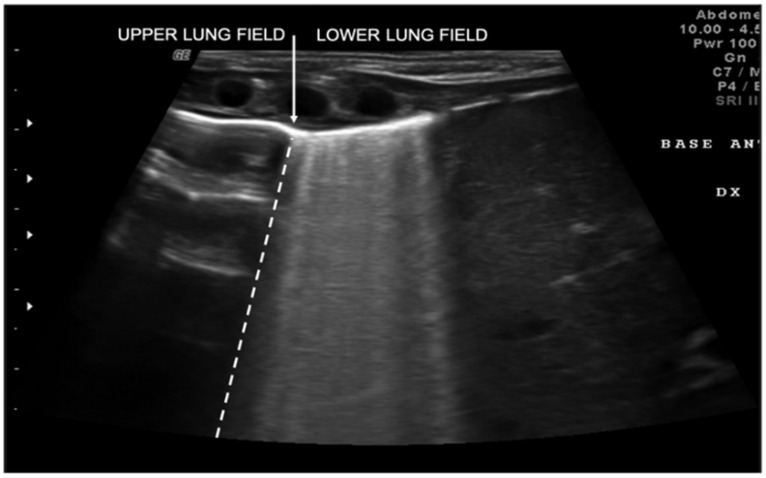
Transient tachypnea of the newborn. The dashed line shows the ultrasound demarcation line between the upper and lower lung fields: double-lung point (white arrow).

Transient tachypnea of the newborn (TTN), also known as “wet lung,” is caused by a failure in the reabsorption of fluid from the fetal lung. It is typical of term or post-term infants in the case of rapid delivery or cesarean section [24]. Infants with TTN present compact B-lines in the lower lung fields and fewer and less compact B-lines in the upper fields in one or both lungs. These signs, also known as double-lung points, appear because of the greater involvement of the lower lung fields in the disease and are characterized by a sharp ultrasound demarcation line between the upper and lower lung fields of both lungs (Figure 5) [17]. The pleural line is regular, with normal echogenicity and movement with respiratory acts. In contrast to NRDS, no subpleural consolidations are observed. Due to these typical ultrasonographic findings, much evidence has shown how pulmonary ultrasonography can be useful in the early diagnosis of TTN and differentiate TTN from NRDS already by the first hours of life [25].

Meconium aspiration syndrome (MAS) refers to a multitude of respiratory symptoms caused by meconium aspiration during delivery. It is more frequent in post-term newborns. The ultrasound findings of MAS are as follows: B-pattern appearance, with coalescent or noncoalescent B-lines, several subpleural consolidations asymmetrically spread on both lungs with few spared areas and in severe cases of MAS a white lung pattern [26].

## 6. Congenital Pulmonary Airway Malformations and Congenital Diaphragmatic Hernia

Congenital malformations of the lung and chest, such as congenital pulmonary airway malformation (CPAM) or congenital diaphragmatic hernia, are usually diagnosed prenatally with ultrasound and with MRI [27]. However, these conditions may be undetected in countries with a lack of prenatal care until the onset of the symptoms after birth. Moreover, the diagnostic accuracy of prenatal ultrasound, even when supported by fetal MRI, is not absolute [28]. Recently, lung ultrasound was proposed as a support in the evaluation of a newborn with respiratory distress in whom CPAM is suspected or to confirm an antenatal suspicion of CPAM [29].

**Figure 6:**
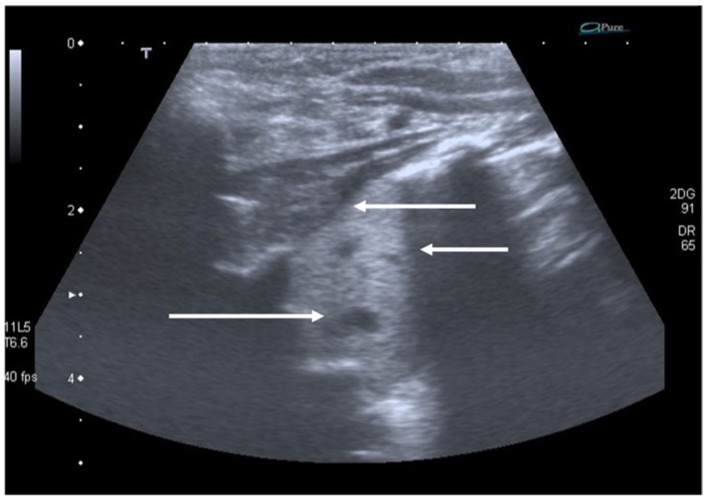
Congenital pulmonary airway malformation. Hepatization is targeted by the upper arrows. The lower arrow highlights the wider anechogenic round cystic areas.

The typical ultrasonographic appearance of CPAM is not yet well defined or univocal, but a large hypoechogenic cystic single lesion, small communicating cystic lesions and consolidations similar to those observed in other lung diseases have been described in several patients (Figure 6). The first two findings, however, appear to be detectable only in this disease [29]. To date, the gold standard for the postnatal diagnosis of these malformations remains the CT of the chest, but ultrasound may in the future represent a useful test to confirm an uncertain diagnosis or follow the patient over time.

As proposed by Corsini et al., pulmonary ultrasound could contribute to the correct diagnosis in congenital diaphragmatic hernia, even faster than chest X-ray, allowing faster treatment and improving the prognosis [30]. The same authors have reported for the first time the ultrasound features of this congenital pathology: lack of complete visualization of the hyperechogenic line representing the diaphragm an absence of an A-line, a pleural line and lung sliding in the involved area and hyperechogenic moving material typical of the ultrasound image of the gut.

## 7. Respiratory Infectious Diseases in Children

The most frequent respiratory diseases in children are bronchiolitis and community-acquired pneumonia (CAP). The diagnosis of these diseases is mainly clinical, and chest X-ray is recommended only in severe clinical conditions or uncertain diagnoses [31,32].

Recently, some authors focused on the application of ultrasound in these children, with promising data [33,34,35,36], and a consensus of experts in 2020 well established the role of point-of-care ultrasound in the management of bronchiolitis and pneumonia in the pediatric population [37].

**Figure 7:**
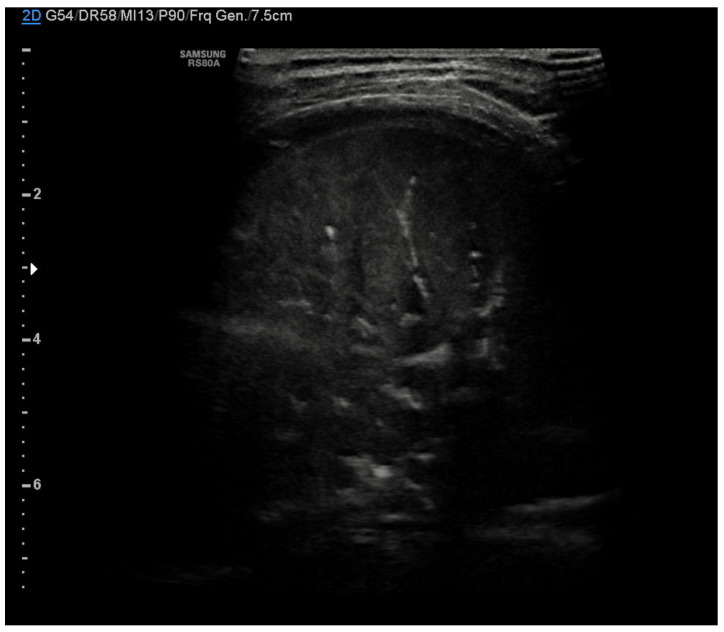
Pneumonia. Hypoechoic areas with a liver pattern inside configuring a consolidation. Air bronchogram is visible.

In community-acquired pneumonia (CAP), the main ultrasound findings are consolidations that appear as hypoechoic areas with evidence of an air bronchogram within and an ecostructural “liver pattern,” with clear margins (indicating a lobar consolidation) or surrounded by confluent B-lines (Figure 7) [6].

The liver-like ultrasound appearance, which defines the hepatization of the lung, is consequent to the filling of the alveoli with an inflammatory and purulent material that determines a solid and isoechogenic appearance of the lung. Frequently within the consolidations, it is possible to observe a fine hyperechogenic arborescent pattern that defines the air bronchogram due to the presence of air remaining inside the small bronchioles. The air bronchogram typical of pneumonic consolidations is defined as dynamic due to the possibility of observing the air move back and forth with the breaths, a testament to their patency. This ultrasound sign differs from the static air bronchogram, in which air movement is not observable, typical of atelectasis [38].

Sometimes, detecting large consolidations can be difficult, especially in overweight patients. Recently, Møller-Sørensen et al. described an ultrasound finding that may be helpful in detecting larger consolidations, where a thinner and less bright pleural line can be observed when the underlying lung tissue is consolidated [39].

LUS is a valuable method for the monitoring of lung consolidation in children, avoiding frequent exposure to a massive dose of ionizing radiation [40,41,42]. With the resolution of the infectious process, it is possible to observe changes in the size of the lung consolidation and bronchogram distribution. In particular, air bronchograms are seen more peripherally because the lung starts to re-expand, determining greater air movement into the consolidated lung. Furthermore, analyzing specific ultrasound features, it is possible to assume different etiologies of CAP in children. Signs of viral CAP than bacterial CAP are multiple consolidations, smaller and often bilateral. Certainly, it is important to interpret these findings in light of clinical and laboratory findings [43,44].

Furthermore, it is possible to detect early complications (as pleural effusion or pneumothorax), making ultrasound an excellent tool for monitoring and follow-up the disease [40]. Currently, many studies demonstrated high sensitivity and specificity of LUS in diagnosis and follow-up of CAP compared to CXR [45,46], and the 2020 consensus established at least the same diagnostic value of LUS to CXR in detecting pneumonia, suggesting ultrasound as the method of choice in children with suspected pneumonia requiring diagnostic imaging [37].

**Figure 8:**
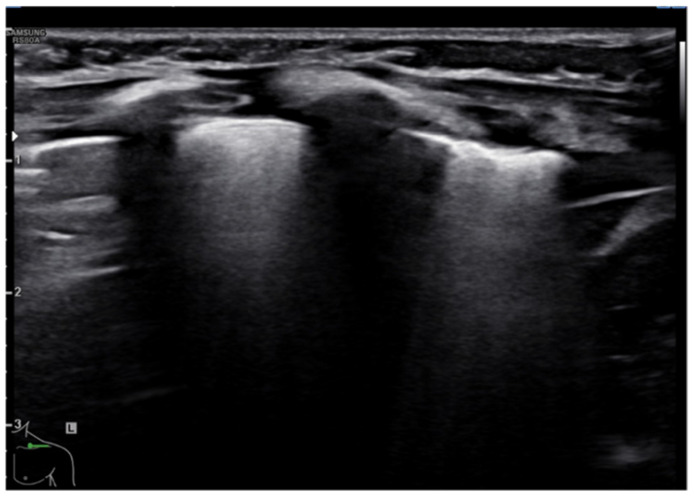
Bronchiolitis. Coalescent B-lines arising from the pleural line.

**Figure 9:**
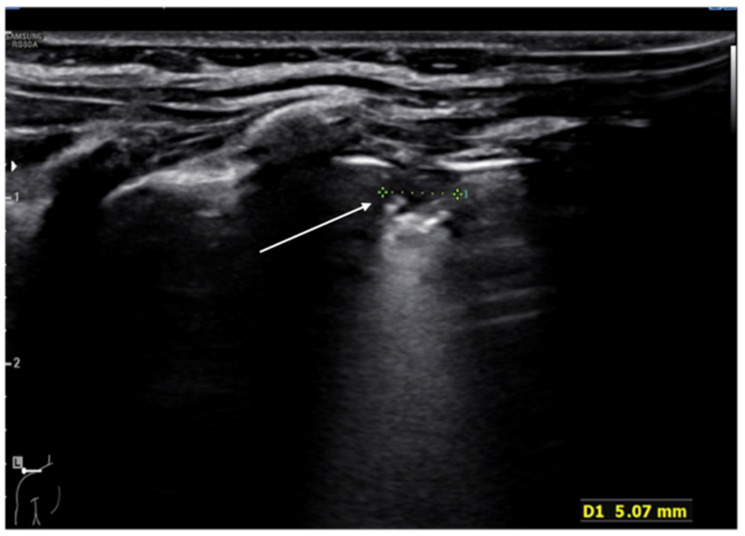
Bronchiolitis. Subpleural consolidation (white arrow).

In bronchiolitis, the main ultrasound findings are: areas of subpleural consolidations defined as hypoechogenic areas with parenchymatous appearance and irregular margins, the presence of coalescent B-lines (three or more B-lines in each intercostal space) up to the “white lung” or focal presence of multiple B-lines (one or two B-lines in each intercostal space) and abnormalities of the pleural line (Figure 8 and Figure 9) [47]. Several studies demonstrated that lung ultrasound findings strictly correlate with the clinical evaluations in infants with bronchiolitis [48,49], and the 2020 consensus established LUS as a useful tool in assessing the severity of the disease [37]. Therefore, LUS could be used as support of the clinical examination in the identification of infants who may require more intensive care as supplementary oxygen or respiratory support [36].

## 8. Pneumothorax, Pleural Effusion Empyema and Lung Abscess

The use of LUS for the diagnosis of pneumothorax dates back to 1995 [15]. Since then, pulmonary ultrasound has been used more and more, flanking the CXR in both medical and trauma patients. In fact, the sensitivity of CXR for pneumothorax is low because air tends to accumulate in the anterior-medial and apical areas of the chest, which are difficult to explore by CXR but can be well assessed by ultrasound [50]. The typical LUS sign of a pneumothorax is the disappearance of lung sliding due to the presence of air in the pleural cavity [15]. A second sign is also represented by the absence of B-lines, while some horizontal artifactual lines reverberating distal to the probe and similar to the A-lines can usually be observed. An additional ultrasound sign, the so-called “lung point,” which is highly specific for pneumothorax, is the exact point of transition between the presence and absence of sliding. At this interface, it will be possible to observe a normal lung sliding in the healthy regions of the thorax and the absence of the sliding in those areas adjacent to the pneumothorax [51]. The same ultrasound signs can also be observed in newborns in case of air leak syndrome, of which pneumothorax is the most frequent type and seen in approximately 2–10% of very-low-birthweight patients [52].

**Figure 10:**
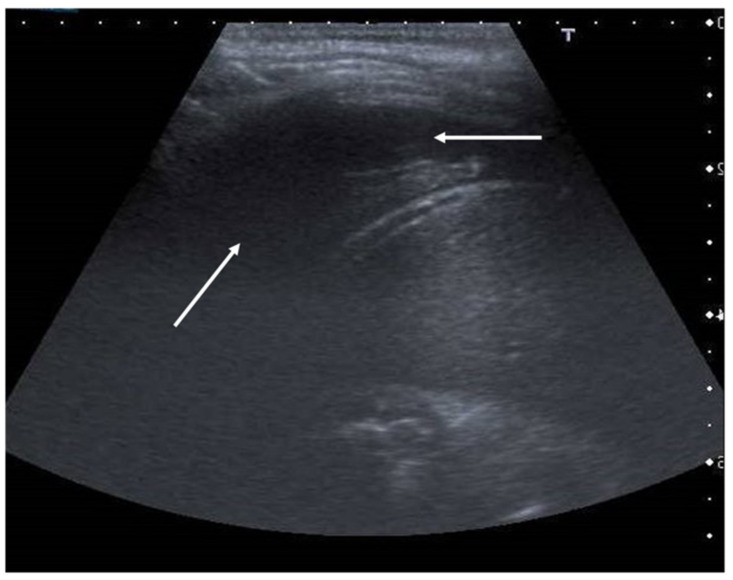
Anechogenic fluid collection above the diaphragm suggestive of pleural effusion (white arrows).

The role of LUS in identifying pleural effusion is also well established [53]. In fact, many studies have evaluated the efficacy of lung ultrasound in the diagnosis of pleural effusion, calculating its sensitivity and specificity, which have often been close to 100% [54]. These studies have confirmed the superiority of ultrasound over CXR in pleural effusion detection due to the capability of ultrasound to detect fluid collections as small as 20 mL [55]. Usually, pleural effusion can be visualized by ultrasound as a dark and anechogenic region located above the diaphragm that also determines the disappearance of the mirror image of the liver and spleen in the lung fields, which can be visualized in the healthy lung (Figure 10) [14]. Ultrasound also allows quantifying the amount of fluid accumulated in the pleural cavity well and characterizing the fluid collection differentiating a transudate from an exudate. The transudate is characterized by a dark image without internal echoes, representing an uncomplicated collection, while an image characterized by a septate or multiloculated corpuscular fluid collection is typical of the exudate [14].

**Figure 11:**
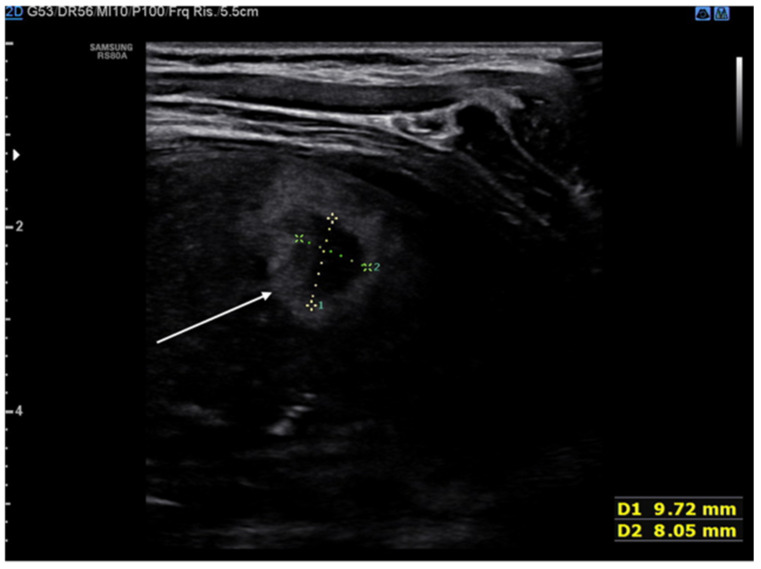
Hypoechogenic capsule lesion with irregular, hyperechogenic edges suggestive of lung abscess (white arrow).

The lung abscess appears as a well-demarcated capsular structure surrounding a hypoechoic core without internal vascularity on color Doppler (Figure 11). Furthermore, it is possible to distinguish lung abscess from pyopneumothorax through the presence of all four specific signs: air-fluid level, synchronous movement of air-fluid levels with breaths, loss of gliding sign above the air-fluid level and the suspended microbubble sign (punctate hyperechoic pinpoints with shadows that move more or less randomly with respiratory movement within the pleural effusion) [56,57].

**Figure 12:**
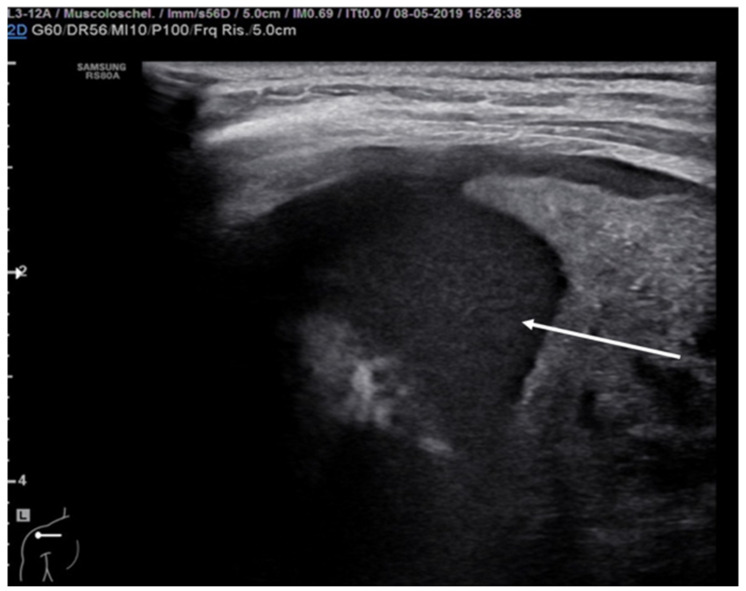
Hypoechogenic and highly corpuscular collection suggestive of pleural empyema (white arrow).

Pleural empyema refers to an infected, purulent and often loculated pleural effusion due to parenchymal infection that spreads to the pleural cavity. Ultrasound examination of the chest revealed a hypoechoic lesion with complex-septated effusions, passive atelectasis, width uniformity and smooth luminal and outer margins (Figure 12). Color Doppler can be used to differentiate the peripheral air-fluid abscess from empyema. In fact, color Doppler ultrasound vessel signals in pericavitary consolidation are a predictor of lung abscess [57,58,59,60].

Due to the ability of ultrasonography to investigate the pleural space, estimate the amount of effusion and describe its features, ultrasonography is not only a useful tool for differentiating pleural effusion from empyema and abscess but can also play an important role in the operative decision to drain or not an effusion or to instill fibrinolytic agents in the pleural space or even to resort to more invasive surgical procedures [61]. Although, to date, there are no evidence-based criteria to make the indication for surgery in the pediatric patient, failure of chest tube drainage, antibiotics and fibrinolysis appear to be possible indications as well as a persistent septic state in association with a pleural collection, despite chest tube drainage and antibiotic therapy [62]. Furthermore, ultrasound is most frequently used to evaluate or guide aspiration and drain insertion in patients with suspected pleural effusions. In fact, increased success rates have been reported evaluating real-time US-guided chest drain insertion. US guidance allows constant monitoring with complete visualization of the needle tip, thus minimizing the risk of drain misplacement or visceral or lung damage [61,62,63].

## 9. COVID-19 Pneumonia

After the outbreak of the severe acute respiratory syndrome coronavirus 2 (SARS-CoV-2) pandemic, the rapid diagnosis of COVID-19 interstitial pneumonia, its management and treatment have become pivotal. In the case of COVID-19 pneumonia, the gold standard for the diagnosis and monitoring of the disease is considered computer tomography (CT) [64]. However, this technique is expensive, not universally available, uses a dose of ionizing radiation and requires moving the patient to another department with the potential risk of exposure for health care workers. For these reasons, LUS that can be performed at the patient’s bedside could be a promising tool for diagnosis and management of COVID-19 pneumonia.

SARS-CoV-2 infection in the pediatric population appears to be less severe than in the adult population, and in most cases, the disease is mild or moderate [65]. Despite this, pulmonary involvement or SARS-CoV-2 pneumonia has also been described in asymptomatic or poorly symptomatic children [66].

To date, the sonographic appearance of SARS-CoV-2 infection has been best described in adult patients. In adults, COVID-19 pneumonia LUS demonstrates bilateral lung involvement with the typical interstitial syndrome pattern characterized by isolated/multiple or coalescent B-lines, sometimes separated by spared areas, a thinned pleural line with some irregularities, and sometimes pleural effusion and subpleural consolidations [67]. B-lines, the number of which increases as the air content in the lung decreases, are considered a typical sign of the interstitial syndrome and their distribution seems to correlate with the severity of the clinical picture [68].

Recently, much evidence has also encouraged the use of lung ultrasound in the pediatric population by describing the ultrasound findings of SARS-CoV-2 infection in children. In particular, Musolino et al. described in a cohort of 10 pediatric patients ultrasonographic findings similar to those observed in adults, with bilateral lung involvement in 70% of cases, pleural line irregularities in 60% of cases and subpleural consolidations in 10% of cases. In addition, vertical reverberation artifacts were observed in 70% of patients. In contrast, pleural effusion was not observed in any patient [69]. In an even more recent study conducted on a wider cohort of patients, Musolino et al. found the same ultrasound findings as described above, reporting that children with moderate disease had a statistically significant increase in the number of B-lines. The same study also showed that at 96 h after the first lung ultrasound, only 20% of the children had ultrasound abnormalities, with a statistically significant reduction in pleural line irregularities and B-line [70].

Similar ultrasonographic findings were reported in a study by Denina et al. on a cohort of eight children. Subpleural consolidations were observed in two cases, whereas confluent B-lines were observed in five of the eight patients [7]. In addition, the same study highlighted the concordance of lung ultrasound with chest X-ray in seven/eight patients, whereas, in one, the ultrasound showed a pattern of interstitial B-lines despite normal chest radiography.

However, neither of the two studies cited above demonstrated a correlation between ultrasound appearance and the severity of the disease. However, as suggested by Norbedo et al., LUS could play an important role as a triage tool in children with paucisymptomatic and mild forms of the disease and in the management of patients with a negative nasal swab but with ultrasonographic findings suggestive of disease to recommend precautionary isolation of the patient pending repeat nasal swab [71].

## 10. Diagnosis and Follow-Up of Pulmonary Edema

Acute pulmonary edema is characterized by accumulation of the fluid in alveoli and interstitial spaces. Its diagnosis with lung ultrasound is increasingly widespread and useful, especially when a CT scan cannot be used or when a rapid bedside examination is necessary. The artifact B-line was used for the detection of extravascular lung water. Different ultrasound scanning protocols are described to evaluate pulmonary edema [72,73,74,75]. The normal lung is black (no signal), the abnormal wet lung with interstitial pulmonary edema is black and white (with focal B-lines) and the lung with alveolar pulmonary edema is white (confluent B-lines in a fully echogenic lung). The number of B-lines in the anterolateral chest scan is usually summed to generate a quantitative or semiquantitative B-line score (≤5, extravascular lung water absent; 6–15, mild degree; 16–30, moderate degree; >30, severe degree) [76].

## 11. Thoracic Trauma and Detection of Signs of Child Abuse

Ultrasound plays a primary role in the management of the polytrauma patient. In fact, because hemorrhage is the first cause of mortality and morbidity in trauma patients, the use of a rapid technique such as ultrasound to investigate the presence of hemorrhage in the pericardial or pleural cavity, as well as in the peritoneal cavity, represents today a milestone in the primary Advanced Trauma Life Support (ATLS) survey [77].

Since the mid-2000s, an extension of the Focused Assessment with Sonography for Trauma (FAST) protocol, E-FAST (Extended-FAST), has been developed, aimed at extending the evaluation, previously limited to the heart and abdominal wall, to the thoracic cavity, to search for pleural effusion and pneumothorax [78]. Thanks to progressively better-defined semeiotics of the healthy and pathological lung, today, ultrasound represents an irreplaceable tool in the emergency department with a higher sensitivity for pneumothorax and hemothorax than CXR [15,79].

**Figure 13:**
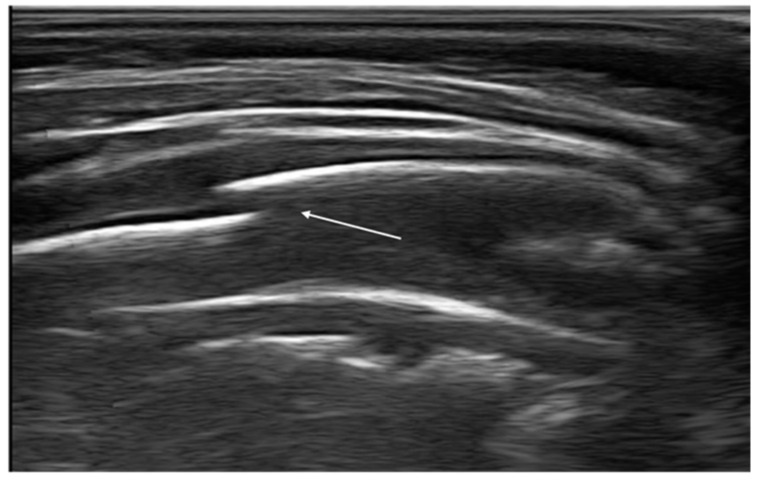
Rib fracture in transverse scan along the major axis of the rib (white arrow).

In addition, recent evidence has shown that ultrasounds may be a useful tool for the detection of bone fractures, in particular rib fractures [80]. A rib fracture is diagnosed when a discontinuity of the cortical alignment is observed as a clear disruption of the anterior echogenic margin of the rib (Figure 13). Furthermore, in the case of previous fractures, it is possible to observe at the ultrasound the bone callus that determines an irregular cortical profile different from that of the adjacent ribs. Rib fractures are suspected in pediatric age; in fact, they represent indicators of child abuse. Therefore, thoracic ultrasound could be helpful in identifying, promptly, children at risk of abuse, reducing the exposure to ionizing radiation [81,82].

## 12. Follow-Up in Pediatric Patients Undergoing Cardiac Surgery

Recently, several studies have described the role of LUS as a complementary tool for CXR in the perioperative evaluation of children undergoing cardiac surgery [83,84]. LUS may provide not only diagnostic but also prognostic information in a pediatric cardiac surgery setting. Primarily, the methodic can be useful to evaluate the degree of pulmonary congestion that is a frequent complication after pediatric cardiac surgery. In children, there is no standardized system to classify pulmonary congestion according to the percentage of B-lines. The classification proposed by Raimondi et al. [85] is more simple than an adult scoring system and includes four grades: 1—no B lines; 2—few B lines; 3—moderate B lines; 4—severe B lines. Some authors described the correlation between LUS profiles and clinical outcomes as cardiopulmonary bypass duration, aortic clamp time and time of extubation and intensive care unit stay [86]. LUS may be employed for the diagnosis of many common lung complications occurring after cardiac surgery, including atelectasis, effusion, pneumonia, pneumothorax and diaphragmatic motion anomalies, and guide interventional procedures such as drainage insertion for pleural effusion and pneumothorax [87,88,89]. Another application is the diagnosis of retrosternal clots after pediatric cardiac surgery that are often difficult to see with conventional echocardiography [90].

Furthermore, some authors have suggested the role of lung ultrasound in the evaluation of the effects of the incremental PEEP recruitment maneuver in children undergoing cardiac surgery in the reduction of atelectasis, improved lung aeration, oxygenation and respiratory system dynamic compliance for kilogram body weight [91].

## 13. Diaphragm Ultrasound

Ultrasound can be useful to assess diaphragmatic function. In adult patients, there are many clinical indications for ultrasonography of the diaphragm: diagnosis and monitoring of diaphragmatic paralysis, neuromuscular disorders, guidance for needle electromyography, assessment in chronic diseases, traumatic diaphragm rupture, detection of postoperative complication, difficult weaning, estimating work of breathing and titrating ventilatory support [92].

Recently, different studies have evaluated the application of diaphragmatic ultrasound in pediatric age. In particular, some have authors investigated the predictive value of diaphragm ultrasound for weaning outcomes in critically ill children. In particular, the diaphragmatic parameters with better performance were diaphragmatic thickening fraction and maximum inspiratory pressure [93].

Furthermore, an interesting application is that described by Buonsenso et al. The authors showed diaphragmatic US findings in infants with bronchiolitis and they found a correlation between ultrasound diaphragm parameters (diaphragm excursion, inspiratory excursion, thickness at end-inspiration) and clinical outcomes [94].

## 14. New Perspectivations of LUS

Recently, some technical innovations have been introduced regarding lung ultrasound that could make the method even more informative and simplify its interpretation. Moshavegh et al., for example, have proposed an algorithm for the automatic detection of B-lines able to discriminate between ultrasound images of healthy volunteers and patients undergoing cardiac surgery. This innovative method could in the future be used for a better qualitative and quantitative description of B-lines, useful in patients with interstitial lung disease or pulmonary edema [95].

Ultrasound elastography, previously used mainly for the exploration of the liver or other parenchymatous organs, has also been validated for the examination of the lung. This technique allows evaluating the mechanical characteristics of a tissue that may change in case of disease. To date, some evidence has described the possible role of ultrasound elastography in pathologies such as interstitial lung disease, in the evaluation of solid lesions of the lung and pulmonary edema or pleural effusion [96]. In this sense, Zhang et al. developed a lung ultrasound surface wave elastography technique validating this method first in murine models and then in patients with interstitial lung disease and healthy controls. The results of this study showed that patients with interstitial lung disease had a statistically higher surface wave speed than healthy controls due to lung fibrosis and that, therefore, this imaging technique may be useful in detecting patients with interstitial lung disease [97,98]. In addition, Clay et al. recently demonstrated how the surface wave speed correlates positively with the degree of pulmonary involvement [99].

## 15. Conclusions

Thanks to growing evidence, the semeiotics of the healthy and pathological lung is better characterized, and ultrasound increasingly is used in the diagnosis, management, and follow-up of lung diseases in children and infants. Lung ultrasound is rapidly becoming a valuable tool in the hands of the pediatrician, especially in an emergency department setting. The rapidity, repeatability, low cost and possibility to be performed bedside represent the strengths of this method.

The spread of this method could determine many benefits, contributing to better use of the economic resources of the health system and to the more prudent management of children that avoid radiation exposure, during diagnosis and follow-up of lung and thoracic diseases.

## Data Availability

Data sharing not applicable.

## References

[1] Thomas K.E., Parnell-Parmley J.E., Haidar S., Moineddin R., Charkot E., Bendavid G., Krajewski C. (2006). Assessment of radiation dose awareness among pediatricians. Pediatr. Radiol..

[2] Zechner P.M., Seibel A., Aichinger G., Steigerwald M., Dorr K., Scheiermann P., Schellhaas S., Cuca C., Breitkreutz R., Arbeitsgruppe des Moduls 5 in Anästhesie Fokussierte Sonographie der DGAI (2012). Lung ultrasound in acute and critical care medicine. Anaesthesist.

[3] Volpicelli G., Elbarbary M., Blaivas M., Lichtenstein D.A., Mathis G., Kirkpatrick A.W., Melniker L., Gargani L., Noble V.E., Via G. (2012). International evidence-based recommendations for point-of-care lung ultrasound. Intensive Care Med..

[4] Joshi P., Vasishta A., Gupta M. (2019). Ultrasound of the pediatric chest. Br. J. Radiol..

[5] Cattarossi L. (2013). Lung ultrasound: Its role in neonatology and pediatrics. Early Hum. Dev..

[6] Copetti R., Cattarossi L. (2008). Ultrasound diagnosis of pneumonia in children. Radiol. Med..

[7] Denina M., Scolfaro C., Silvestro E., Pruccoli G., Mignone F., Zoppo M., Ramenghi U., Garazzino S. (2020). Lung ultrasound in children with COVID-19. Pediatrics.

[8] Feng X.Y., Tao X.W., Zeng L.K., Wang W.Q., Li G. (2020). Application of pulmonary ultrasound in the diagnosis of COVID-19 pneumonia in neonates. Zhonghua Er Ke Za Zhi.

[9] Reissig A., Copetti R. (2014). Lung ultrasound in community-acquired pneumonia and in interstitial lung diseases. Respiration.

[10] Platz E., Jhund P.S., Girerd N., Pivetta E., McMurray J.J.V., Peacock W.F., Masip J., Martin-Sanchez F.J., Miró Ò., Price S. (2019). Study Group on Acute Heart Failure of the Acute Cardiovascular Care Association and the Heart Failure Association of the European Society of Cardiology. Expert consensus document: Reporting checklist for quantification of pulmonary congestion by lung ultrasound in heart failure. Eur. J. Heart Fail..

[11] Soldati G., Smargiassi A., Inchingolo R., Buonsenso D., Perrone T., Briganti D.F., Perlini S., Torri E., Mariani A., Mossolani E.E. (2020). Proposal for international standardization of the use of lung ultrasound for patients with COVID-19. A simple, quantitative, reproducible method. J. Ultrasound Med..

[12] Nenna R., Iovine E., Laudisa M., Bloise S., La Regina D.P., Midulla F. (2021). Comment on Jaworska, J.; et al. Consensus on the Application of Lung Ultrasound in Pneumonia and Bronchiolitis in Children. Diagnostics.

[13] Jaworska J., Komorowska-Piotrowska A., Pomiećko A., Wiśniewski J., Woźniak M., Littwin B., Kryger M., Kwaśniewicz P., Szczyrski J., Kulińska-Szukalska K. (2021). Response to Comment on Jaworska, J.; et al. Consensus on the Application of Lung Ultrasound in Pneumonia and Bronchiolitis in Children. Diagnostics.

[14] Lobo V., Weingrow D., Perera P., Williams S.R., Gharahbaghian L. (2014). Thoracic ultrasonography. Crit. Care Clin..

[15] Lichtenstein D., Menu Y. (1995). A bedside ultrasound sign ruling out pneumothorax in the critically ill. Lung sliding. Chest.

[16] Lichtenstein D., Meziere G., Biderman P., Gepner A., Barré O. (1997). The comet-tail artifact. An ultrasound sign of alveolar interstitial syndrome. Am. J. Respir. Crit. Care Med..

[17] Copetti R., Cattarossi L. (2007). The “double lung point”: An ultrasound sign diagnostic of transient tachypnea of the newborn. Neonatology.

[18] Buonsenso D., Soldati G., Curatola A., Morello R., De Rose C., Vacca M.E., Lazzareschi I., Musolino A.M., Valentini P. (2020). Lung Ultrasound Pattern in Healthy Infants during the First 6 Months of Life. J. Ultrasound Med..

[19] Lichtenstein D., Mauriat P. (2012). Lung ultrasound in the critically ill neonate. Curr. Pediatr. Rev..

[20] Sharma D., Farahbakhsh N. (2019). Role of chest ultrasound in neonatal lung disease: A review of current evidences. J. Matern. Fetal Neonatal Med..

[21] Copetti R., Cattarossi L., Macagno F., Violino M., Furlan R. (2008). Lung ultrasound in respiratory distress syndrome: A useful tool for early diagnosis. Neonatology.

[22] Sweet D.G., Carnielli V., Greisen G., Hallman M., Ozek E., Pas A.T., Plavka R., Roehr C.C., Saugstad O.D., Simeoni U. (2019). European Consensus Guidelines on the Management of Respiratory Distress Syndrome—2019 Update. Neonatology.

[23] Gregorio-Hernández R., Arriaga-Redondo M., Pérez-Pérez A., Ramos-Navarro C., Sánchez-Luna M. (2020). Lung ultrasound in preterm infants with respiratory distress: Experience in a neonatal intensive care unit. Eur. J. Pediatrics.

[24] Reuter S., Moser C., Baack M. (2014). Respiratory distress in the newborn. Pediatr. Rev..

[25] Liu J., Wang Y., Fu W., Yang C.S., Huang J.J. (2014). Diagnosis of neonatal transient tachypnea and its differentiation from respiratory distress syndrome using lung ultrasound. Medicine.

[26] Piastra M., Yousef N., Brat R., Manzoni P., Mokhtari M., De Luca D. (2014). Lung ultrasound findings in meconium aspiration syndrome. Early Hum. Dev..

[27] David M., Lamas-Pinheiro R., Henriques-Coelho T. (2016). Prenatal and postnatal management of congenital pulmonary airway malformation. Neonatology.

[28] Farrugia M., Raza S., Gould S., Lakhoo K. (2008). Congenital lung lesions: Classification and concordance of radiological appearance and surgical pathology. Pediatr. Surg. Int..

[29] Yousef N., Mokhtari M., Durand P., Raimondi F., Migliaro F., Letourneau A., Tissières P., De Luca D. (2018). Lung Ultrasound Findings in Congenital Pulmonary Airway Malformation. Am. J. Perinatol..

[30] Corsini I., Parri N., Coviello C., Leonardi V., Dani C. (2019). Lung ultrasound findings in congenital diaphragmatic hernia. Eur. J. Pediatr..

[31] Harris M., Clark J., Coote N., Fletcher P., Harnden A., Mckean M., Thomson A. (2011). British Thoracic society guidelines for the management of community acquired pneumonia in children: Update 2011. Thorax.

[32] Bradley J., Byington C., Shah S. (2011). The management of community-acquired pneumonia in infants and children older than 3 months of age: Clinical practice guidelines by the Pediatric Infectious Diseases Society and the Infectious Diseases Society of America. Clin. Infect. Dis..

[33] Balk D.S., Lee C., Schafer J., Welwarth J., Hardin J., Novack V., Yarza S., Hoffmann B. (2018). Lung ultrasound compared to chest-X-ray for diagnosis of pediatric pneumonia: A meta-analysis. Pediatr. Pulmonol..

[34] Stadler J., Andronikou S., Zar H. (2017). Lung ultrasound for the Diagnosis of Community-Acquired Pneumonia in children. Pediatr. Radiol..

[35] Supino M.C., Buonsenso D., Scateni S., Scialanga B., Mesturino M.A., Bock C., Chiaretti A., Giglioni E., Reale A., Musolino A.M. (2019). Point-of-care lung ultrasound in infants with bronchiolitis in the pediatric emergency department: A prospective study. Eur. J. Pediatr..

[36] Bueno-Campaña M., Sainz T., Alba M., Del Rosal T., Mendez-Echevarría A., Echevarria R., Tagarro A., Ruperez-Lucas M., Herrreros M.L., Latorre L. (2019). Lung ultrasound for prediction of respiratory support in infants with acute bronchiolitis: A cohort study. Pediatr. Pulmonol..

[37] Jaworska J., Komorowska-Piotrowska A., Pomiećko A., Wiśniewski J., Woźniak M., Littwin B., Kryger M., Kwaśniewicz P., Szczyrski J., Kulińska-Szukalska K. (2020). Consensus on the Application of Lung Ultrasound in Pneumonia and Bronchiolitis in Children. Diagnostics.

[38] Lichtenstein D., Mezière G., Seitz J. (2009). The dynamic air bronchogram. A lung ultrasound sign of alveolar consolidation ruling out atelectasis. Chest.

[39] Møller-Sørensen H., Gjedsted J., Jørgensen V.L., Hansen K.L. (2020). COVID-19 Assessment with Bedside Lung Ultrasound in a Population of Intensive Care Patients Treated with Mechanical Ventilation and ECMO. Diagnostics.

[40] Urbankowska E., Krenke K., Drobczyński Ł., Korczyński P., Urbankowski T., Krawiec M., Kraj G., Brzewski M., Kulus M. (2015). Lung ultrasound in the diagnosis and monitoring of community acquired pneumonia in children. Respir. Med..

[41] Reissig A., Copetti R., Mathis G., Mempel C., Schuler A., Zechner P., Aliberti S., Neumann R., Kroegel C., Hoyer H. (2012). Lung ultrasound in the diagnosis and follow-up of community-acquired pneumonia: A prospective, multicenter, diagnostic accuracy study. Chest.

[42] Buonsenso D., Brancato F., Valentini P., Curatola A., Supino M., Musolino A.M. (2020). The Use of Lung Ultrasound to Monitor the Antibiotic Response of Community-Acquired Pneumonia in Children: A Preliminary Hypothesis. J. Ultrasound Med..

[43] Berce V., Tomazin M., Gorenjak M. (2019). The Usefulness of Lung Ultrasound for the Aetiological Diagnosis of Community-Acquired Pneumonia in Children. Sci. Rep..

[44] Kharasch S., Duggan N., Cohen A. (2020). Lung Ultrasound in Children with Respiratory Tract Infections: Viral, Bacterial or COVID-19? A Narrative Review. Open Access Emerg. Med..

[45] Bloise S., La Regina D., Pepino D., Iovine E., Laudisa M., Di Mattia G., Nicolai A., Nenna R., Petrarca L., Mancino E. (2020). Lung ultrasound compared to chest X-ray for the diagnosis of CAP in children. Pediatr. Int..

[46] Claes A.-S., Clapuyt P., Menten R., Michoux N., Dumitriu D. (2017). Performance of chest ultrasound in pediatric pneumonia. Eur. J. Radiol..

[47] Caiulo V.A., Gargani L., Caiulo S., Fisicaro A., Moramarco F., Latini G., Picano E. (2011). Lung ultrasound in bronchiolitis: Comparison with chest X-ray. Eur. J. Pediatr..

[48] La Regina D.P., Bloise S., Pepino D., Iovine E., Laudisa M., Cristiani L., Nicolai A., Nenna R., Mancino E., Di Mattia G. (2021). Lung ultrasound in bronchiolitis. Pediatr. Pulmonol..

[49] Özkaya A., Yilmaz H., Kendir Ö., Gökay S.S., Eyüboğlu İ. (2020). Lung Ultrasound Findings and Bronchiolitis Ultrasound Score for Predicting Hospital Admission in Children with Acute Bronchiolitis. Pediatr. Emerg. Care.

[50] Dahmarde H., Parooie F., Salarzaei M. (2019). Accuracy of Ultrasound in Diagnosis of Pneumothorax: A Comparison between Neonates and Adults-A Systematic Review and Meta-Analysis. Can. Respir. J..

[51] Lichtenstein D., Mezière G., Biderman P., Gepner A. (2000). The “lung point”: An ultrasound sign specific to pneumothorax. Intensive Care Med..

[52] Boo N., Cheah I. (2011). Malaysian National Neonatal Registry. Risk factors associated with pneumothorax in Malaysian neonatal intensive care units. J. Paediatr. Child Health.

[53] Grimberg A., Shigueoka D., Atallah A.N., Ajzen S., Iared W. (2010). Diagnostic accuracy of sonography for pleural effusion: Systematic review. Sao Paulo Med. J..

[54] Soni N.J., Franco R., Velez M.I., Schnobrich D., Dancel R., Restrepo M.I., Mayo P.H. (2015). Ultrasound in the diagnosis and management of pleural effusions. J. Hosp. Med..

[55] Prina E., Torres A., Carvalho C. (2014). Lung ultrasound in the evaluation of pleural effusion. J. Bras. Pneumol..

[56] Kraft C., Lasure B., Sharon M., Patel P., Minardi J. (2018). Pediatric Lung Abscess Immediate Diagnosis by Point-of-Care Ultrasound. Pediatr. Emerg. Care.

[57] Lin F., Chou C., Chang S. (2004). Differentiating pyopneumothorax and peripheral lung abscess: Chest ultrasonography. Am. J. Med. Sci..

[58] Calder A., Owens C.M. (2009). Imaging of parapneumonic pleural effusions and empyema in children. Pediatr. Radiol..

[59] Svigals P., Chopra A., Ravenel J., Nietert P.J., Huggins J.T. (2017). The accuracy of pleural ultrasonography in diagnosing complicated parapneumonic pleural effusions. Thorax.

[60] Chen H.J., Yu Y.H., Tu C.Y., Chen C.H., Hsia T.C., Tsai K.D., Shih C.M., Hsu W.H. (2009). Ultrasound in peripheral pulmonary air-fluid lesions. Color doppler imaging as an aid in differentiating empyema and abscess. Chest.

[61] De Benedictis F., Kerem E., Chang A.B., Colin A.A., Zar H.J., Bush A. (2020). Complicated pneumonia in children. Lancet.

[62] Balfour-Lynn I.M., Abrahamson E., Cohen G., Hartley J., King S., Parikh D., Spencer D., Thomson A.H., Urquhart D. (2005). Paediatric Pleural Diseases Subcommittee of the BTS Standards of Care Committee. BTS guidelines for the management of pleural infection in children. Thorax.

[63] Lewis M., Micic T., Doull I.J.M., Evans A. (2018). Real-time ultrasound-guided pigtail catheter chest drain for complicated parapneumonic effusion and empyema in children—16-year, single-centre experience of radiologically placed drains. Pediatr. Radiol..

[64] Chung M., Bernheim A., Mei X., Zhang N., Huang M., Zeng X., Cui J., Xu W., Yang Y., Fayad Z.A. (2020). CT imaging features of 2019 novel coronavirus (2019 nCoV). Radiology.

[65] Isoldi S., Mallardo S., Marcellino A., Bloise S., Dilillo A., Iorfida D., Testa A., Del Giudice E., Martucci V., Sanseviero M. (2021). The comprehensive clinic, laboratory, and instrumental evaluation of children with COVID-19: A 6-months prospective study. J. Med. Virol..

[66] Dong Y., Mo X., Hu Y., Qi X., Jiang F., Jiang Z., Tong S. (2020). Epidemiology of COVID-19 among children in China. Pediatrics.

[67] Buonsenso D., Pata D., Chiaretti A. (2020). COVID-19 outbreak: Less stethoscope, more ultrasound. Lancet Respir. Med..

[68] Xing C., Li Q., Du H., Kang W., Lian J., Yuan L. (2020). Lung ultrasound findings in patients with COVID-19 pneumonia. Crit. Care.

[69] Musolino A.M., Supino M.C., Buonsenso D., Ferro V., Valentini P., Magistrelli A., Lombardi M.H., Romani L., D’Argenio P., Campana A. (2020). Roman Lung Ultrasound Study Team for Pediatric COVID-19 (ROMULUS COVID Team). Lung Ultrasound in Children with COVID-19: Preliminary Findings. Ultrasound Med. Biol..

[70] Musolino A.M., Supino M.C., Buonsenso D., Papa R.E., Chiurchiù S., Magistrelli A., Barbieri M.A., Raponi M., D’Argenio P., Villani A. (2021). Lung ultrasound in the diagnosis and monitoring of 30 children with coronavirus disease 2019. Pediatric Pulmonol..

[71] Norbedo S., Blaivas M., Raffaldi I., Caroselli C. (2020). Lung Ultrasound Point-of-View in Pediatric and Adult COVID-19 Infection. J. Ultrasound Med..

[72] Gargani L., Frassi F., Soldati G., Tesorio P., Gheorghiade M., Picano E. (2008). Ultrasound lung comets for the differential diagnosis of acute cardiogenic dyspnoea: A comparison with natriuretic peptides. Eur. J. Heart Fail..

[73] Vitturi N., Soattin M., Allemand E., Simoni F., Realdi G. (2011). Thoracic ultrasonography: A new method for the work-up of patients with dyspnea. J. Ultrasound.

[74] Volpicelli G., Mussa A., Garofalo G., Cardinale L., Casoli G., Perotto F., Fava C., Frascisco M. (2006). Bedside lung ultrasound in the assessment of alveolar-interstitial syndrome. Am. J. Emerg. Med..

[75] Picano E., Frassi F., Agricola E., Gligorova S., Gargani L., Mottola G. (2006). Ultrasound lung comets: A clinically useful sign of extravascular lung water. J. Am. Soc. Echocardiogr..

[76] Picano E., Pellikka P. (2016). Ultrasound of extravascular lung water: A new standard for pulmonary congestion. Eur. Heart J..

[77] Wongwaisayawan S., Suwannanon R., Prachanukool T., Sricharoen P., Saksobhavivat N., Kaewlai R. (2015). Trauma Ultrasound. Ultrasound Med. Biol..

[78] Flato U., Guimarães H., Lopes R.D., Valiatti J.L., Flato E.M., Lorenzo R.G. (2010). Usefulness of Extended-FAST (EFAST-Extended Focused Assessment with Sonography for Trauma) in critical care setting. Rev. Bras. Ter. Intensiva.

[79] Staub L., Biscaro R., Kaszubowski E., Maurici R. (2018). Chest ultrasonography for the emergency diagnosis of traumatic pneumothorax and haemothorax: A systematic review and meta-analysis. Injury.

[80] Yousefifard M., Baikpour M., Ghelichkhani P., Asady H., Darafarin A., Esfahani M.R.A., Hosseini M., Yaseri M., Safari S. (2016). Comparison of ultrasonography and radiography in detection of thoracic bone fractures; a systematic review and meta-analysis. Emergency.

[81] Bloise S., Martucci V., Marcellino A., Mallardo S., Lubrano R. (2020). Possible Role of Thoracic Ultrasound in the Diagnostic Pathway of Infant Abuse in the Pediatric Emergency Department. J. Ultrasound Med..

[82] Battle C., Hayward S., Eggert S., Evans P.A. (2019). Comparison of the use of lung ultrasound and chest radiography in the diagnosis of rib fracture: A systematic review. Emerg. Med. J..

[83] Song I., Kim E., Lee J., Kang P., Kim H.S., Kim J.T. (2018). Utility of perioperative lung ultrasound in pediatric cardiac surgery: A randomized controlled trial. Anesthesiology.

[84] Townsley M. (2020). Lung Ultrasound in Pediatric Cardiac Surgery: A Complementary Tool for Predicting and Identifying Postoperative Pulmonary Complications. J. Cardiothorac. Vasc. Anesth..

[85] Raimondi F., Migliaro F., Sodano A., Umbaldo A., Romano A., Vallone G., Capasso L. (2012). Can neonatal ultrasound monitor fluid clearence and preditc the need of respiratory support?. Crit. Care.

[86] Vitale V., Ricci Z., Gaddi S., Testa G., Toma P., Cogo P. (2017). Lung ultrasound profile after cardiopulmonary bypass in paediatric cardiac surgery: First experience in a simple cohort. Interact. Cardiovasc. Thorac. Surg..

[87] Cantinotti M., Giordano R., Volpicelli G., Kutty S., Murzi B., Assanta N., Gargani L. (2016). Lung ultrasound in adult and paediatric cardiac surgery: Is it time for routine use?. Interact. Cardiovasc. Thorac. Surg..

[88] Vitale V., Ricci Z., Cogo P. (2014). Lung ultrasonography and pediatric cardiac surgery: First experience with a new tool for postoperative lung complications. Ann. Thorac. Surg..

[89] Polito A., Biasucci D., Cogo P. (2016). Point-of-care pleural and lung ultrasound in a newborn suffering from cardiac arrest due to tension pneumothorax after cardiac surgery. Cardiol. Young.

[90] Cantinotti M., Giordano R., Assanta N., Murzi B., Gargani L. (2015). Chest Ultrasound: A New, Easy, and Radiation-Free Tool to Detect Retrosternal Clot after Pediatric Cardiac Surgery. J. Cardiothorac. Vasc. Anesth..

[91] Sun L., Wu L., Zhang K., Tan R., Bai J., Zhang M., Zheng J. (2020). Lung ultrasound evaluation of incremental PEEP recruitment maneuver in children undergoing cardiac surgery. Pediatr. Pulmonol..

[92] Sferrazza Papa G., Pellegrino G., Di Marco F., Imeri G., Brochard L., Goligher E., Centanni S. (2016). A Review of the Ultrasound Assessment of Diaphragmatic Function in Clinical Practice. Respiration.

[93] Xue Y., Zhang Z., Sheng C.-Q., Li Y.-M., Jia F.-Y. (2019). The predictive value of diaphragm ultrasound for weaning outcomes in critically ill children. BMC Pulm. Med..

[94] Buonsenso D., Supino M.C., Giglioni E. (2018). Point of care diaphragm ultrasound in infants with bronchiolitis: A prospective study. Pediatr. Pulmonol..

[95] Moshavegh R., Hansen K.L., Moller-Sorensen H., Nielsen M.B., Jensen J.A. (2019). Automatic Detection of B-Lines in In Vivo Lung Ultrasound. IEEE Trans. Ultrason. Ferroelectr. Freq. Control..

[96] Zhou B., Yang X., Zhang X., Curran W.J., Liu T. (2020). Ultrasound Elastography for Lung Disease Assessment. IEEE Trans. Ultrason. Ferroelectr. Freq. Control..

[97] Zhang X., Osborn T., Zhou B., Brian Bartholmai B., Greenleaf J.F., Kalra S. (2017). An ultrasound surface wave elastography technique for noninvasive measurement of surface lung tissue. J. Acoust. Soc. Am..

[98] Zhang X., Osborn T., Zhou B., Meixner D., Kinnick R.R., Bartholmai B., Greenleaf J.F., Kalra S. (2017). Lung Ultrasound Surface Wave Elastography: A Pilot Clinical Study. IEEE Trans. Ultrason. Ferroelectr. Freq. Control.

[99] Clay R., Bartholmai B., Zhou B., Karwoski R., Peikert T., Osborn T., Rajagopalan S., Kalra S., Zhang X. (2019). Assessment of Interstitial Lung Disease Using Lung Ultrasound Surface Wave Elastography: A Novel Technique with Clinicoradiologic Correlates. J. Thorac. Imaging.

